# Presentation of dizziness in individuals with chronic otitis media: data from the multinational collaborative COMQ-12 study

**DOI:** 10.1007/s00405-021-06993-1

**Published:** 2021-07-21

**Authors:** Bhavesh V. Tailor, John S. Phillips, Ian Nunney, Matthew W. Yung, Can Doruk, Hakan Kara, Taehoon Kong, Nicola Quaranta, Augusto Peñaranda, Daniele Bernardeschi, Chunfu Dai, Romain Kania, Françoise Denoyelle, Tetsuya Tono

**Affiliations:** 1 Department of Otolaryngology, Norfolk and Norwich University Hospitals NHS Foundation Trust, Colney Lane, Norwich, NR4 7UY Norfolk UK; 2 Norwich Clinical Trials Unit, Norwich Medical School, University of East Anglia, Norwich, UK; 3 Department of Otolaryngology, The Ipswich Hospital NHS Trust, Ipswich, UK; 4 Department of Otolaryngology-Head and Neck Surgery, Istanbul University, Istanbul, Turkey; 5 Department of Otolaryngology-Head and Neck Surgery, Yonsei University Wonju College of Medicine, Wonju, Gangwon-do South Korea; 6 Otolaryngology Unit, Department of Biomedical Sciences, Neuroscience and Sensory Organs, University of Bari “Aldo Moro”, Bari, Italy; 7 Fundacion Santa Fe de Bogota, Universidad de Los Andes School of Medicine, Bogota, Colombia; 8 Department of Otolaryngology, Pitié-Salpêtrière Group Hospital, Paris, France; 9 Department of Otology and Skull Base Surgery, Eye and Ear Nose and Throat Hospital, Fudan University, Shanghai, China; 10 Department of Otolaryngology, Lariboisière University Hospital, Paris, France; 11 Department of Otolaryngology-Head and Neck Surgery, Necker-Enfants Malades Hospital, Paris, France; 12 Department of Otolaryngology-Head and Neck Surgery, University of Miyazaki, Miyazaki, Japan

**Keywords:** Otitis Media, Surveys and Questionnaires, Patient-reported outcome measures, Otology, Dizziness, Vertigo

## Abstract

**Purpose:**

In chronic otitis media (COM), disease chronicity and severity of middle ear inflammation may influence the development of inner ear deficits, increasing the risk of vestibular impairment. This secondary analysis of the multinational collaborative Chronic Otitis Media Questionnaire-12 (COMQ-12) dataset sought to determine the prevalence of vestibular symptoms in patients with COM and identify associated disease-related characteristics.

**Methods:**

Adult patients with a diagnosis of COM in outpatient settings at nine otology referral centers across eight countries were included. We investigated the presence of vestibular symptoms (dizziness and/or disequilibrium) using participant responses to item 6 of a native version of the COMQ-12. Audiometric data and otoscopic assessment were also recorded.

**Results:**

This analysis included 477 participants suffering from COM, with 56.2% (*n* = 268) reporting at least mild inconvenience related to dizziness or disequilibrium. There was a significant association between air conduction thresholds in the worse hearing ear and presence of dizziness [adjusted odds ratio (AOR), 1.01; 95% CI 1.00–1.02; *p* = 0.0177]. Study participants in European countries (AOR 1.53; 95% CI 1.03–2.28; *p* = 0.0344) and Colombia (AOR 2.48; 95% CI 1.25–4.92; *p* = 0.0096) were more likely to report dizziness than participants in Asian countries. However, ear discharge and cholesteatoma showed no association with dizziness in the adjusted analyses.

**Conclusion:**

Vestibular symptoms contribute to burden of disease in patients with COM and associates with hearing disability in the worse hearing ear. Geographical variation in presentation of dizziness may reflect financial barriers to treatment or cultural differences in how patients reflect on their health state.

**Supplementary Information:**

The online version contains supplementary material available at 10.1007/s00405-021-06993-1.

## Introduction

Chronic otitis media (COM) is a common disease of the ear, affecting up to 2% of the global population [1]. The overall burden of disease is considerable, especially in the poorest countries [2]. Common sequelae of COM include hearing loss, intermittent or persistent discharge through a perforated eardrum, and tinnitus. This has a negative impact on communication, academia, and employment, resulting in poor quality of life [3, 4].

Dizziness is a common, non-specific, and often untreated complaint in the general population associated with a high degree of handicap and psychological morbidity [5]. It is important to differentiate vestibular vertigo from non-vestibular dizziness since the etiology of dizziness is often multifactorial, especially in elderly patients [6, 7]. Although not the chief complaint, patients with COM frequently report vestibular symptoms including vertigo, postural instability, and disequilibrium [8]. Vestibular function test abnormalities are also commonly detected even in asymptomatic patients, which may indicate central compensation in daily situations [8]. Furthermore, cadaveric studies of human temporal bones have demonstrated significant loss of both cochlear and vestibular hair cells in specimens from donors who had COM, providing histopathological evidence for inner-ear sequelae of chronic middle ear disease [9, 10].

Disease-specific questionnaires assess the impact of specific symptoms and sequelae from the patients’ perspective to enable a comprehensive evaluation of health-related quality of life (HRQoL). Among static instruments for COM, two validated questionnaires contain an item that assesses the degree of inconvenience related to dizziness/balance problems [11, 12]. Specifically, the Chronic Otitis Media Questionnaire-12 (COMQ-12) has undergone an extensive process of psychometric appraisal and cross-cultural adaptation across many countries [11, 13–17], which presents a unique opportunity for comparative research using standardized data. In 2018–2019, a multinational collaborative project was performed, utilizing the COMQ-12 to assess patient-reported HRQoL in nine otology centers across eight countries [18]. This study is based on this dataset. Here, we report the presentation of dizziness in a large cohort of COM patients and identify associated disease-related characteristics.

## Methodology

### Study design

The detailed methods of the multinational collaborative COMQ-12 study has been published previously [18]. In brief, the original study was conducted between 2018 and 2019 at nine otology units across eight countries (China, Colombia, France, Italy, Japan, South Korea, Turkey, and the UK). Eligible participants with COM were prospectively identified during scheduled outpatient consultations. Site leads engaged in local discussions with their Institutional Review Boards, resulting in either formal ethical approval or exemption. Informed patient consent was obtained from all participants prior to study enrolment.

### Participants

Consecutive patients with COM in one or both ears aged 16 years and older were eligible for inclusion and invited to complete a local version of the COMQ-12 questionnaire [11]. The original version of the COMQ-12 in English is provided in *Online Resource 1*. Patients who were unable to comprehend the translated COMQ-12 questionnaire were excluded. Patients did not receive payment or compensation for participation in the study.

### Data collection

A standardized proforma was created using Microsoft Excel^®^ (Microsoft Corporation, Redmond, Washington) and made available to the site leads. Data were initially held on local secure servers at each center adhering to local data governance policies. Patient identifiable data were removed prior to submission to the study lead (J.S.P). Data were collected on: participant demographics; pure-tone air conduction threshold audiometry for both ears (at 0.5, 1.0, 2.0, 3.0, and 4.0 kHz); and otoscopic assessment at point of study recruitment (discharging; perforated tympanic membrane; cholesteatoma).

### Study variables and statistical analyses

As part of the COMQ-12 questionnaire (item 6), participants were asked to score the severity of dizziness or feeling ‘off balance’ over the past 6 months, using a 6-point numeric rating scale (0—no inconvenience; 1—minor inconvenience; 2—moderate inconvenience; 3—major inconvenience but can cope; 4—major inconvenience and difficulty coping; 5—worst thing ever affected life). In the present analysis, the item 6 score was collapsed into a binary dependent variable (> 0 or 0), representing the presence or absence of dizziness, respectively. The following variables were considered as potential disease-related risk factors for self-reported dizziness in patients with COM: worse-ear hearing; ear discharge; and cholesteatoma. Worse-ear hearing refers to the pure-tone average of hearing threshold levels obtained at all assessed frequencies in the poorer hearing ear. The presence of ear discharge and cholesteatoma were reported during otoscopic assessment by the provider.

Identification of significant determinants of dizziness was performed using logistic regression analysis. Odds ratios (ORs) and their 95% confidence intervals (95% CI) were calculated, with both unadjusted and adjusted values (AOR) reported. The level of significance was set at < 0.05. Gender was not recorded for UK study participants and therefore has not been adjusted for in the present analysis. Correcting for surgical intervention was not necessary since less than 1% of recruited participants in the original study had undergone middle-ear surgery historically (i.e., prior to COMQ-12 completion). To model a geographic effect, we used the following region classifiers: *Europe* (France, Italy, Turkey, UK) and *Asia* (China, Japan, Korea). Descriptive statistics and logistic regression modeling were performed using SAS v9.4 (SAS Institute Inc., Cary, NC, USA).

## Results

A total of 478 participants from 8 countries were included in the original study. Of these, 477 participants (99.8%) completed the questionnaire item concerning dizziness or disequilibrium in the COMQ-12 (item 6) and were therefore eligible for inclusion in the present analysis.

Within this cohort, 43.8% (*n* = 209) reported no inconvenience related to dizziness; 16.6% (*n* = 79) reported minor inconvenience, 10.3% (*n* = 49) reported moderate inconvenience and the remaining 29.4% (*n* = 140) reported major inconvenience or greater. Table 1 shows the full descriptive statistics of participants according to the degree of perceived severity of dizziness.

**Table 1:** Distribution of perceived severity of dizziness and associated participant characteristics (*n* = 477)

Perceived dizziness severity^a^	*n* (%)	Mean age (SD)	Mean hearing^b^ (dB, SD)	Overall HRQoL^c^ (SD)
0	209 (43.8)	47.1 (19.1)	30.6 (17.3)	18.7 (10.0)
1	79 (16.6)	51.7 (16.8)	34.8 (19.3)	19.9 (10.2)
2	49 (10.3)	45.6 (17.2)	34.6 (23.0)	27.2 (9.8)
3	60 (12.6)	48.5 (18.2)	29.2 (14.4)	28.9 (9.2)
4	52 (10.9)	47.5 (18.2)	32.0 (17.9)	32.1 (10.5)
5	28 (5.9)	54.0 (17.8)	41.3 (20.3)	38.3 (10.8)

*dB* decibel, *HRQoL* health-related quality-of-life, *SD* standard deviation

^a^Determined by participant responses to item 6 in the Chronic Otitis Media Questionnaire-12 (COMQ-12). Answers were presented using a 6-point Likert scale (0 = no inconvenience, 1 = minor inconvenience, 2 = moderate inconvenience, 3 = major inconvenience but can cope, 4 = major inconvenience and difficulty coping, 5 = worst thing ever affected life)

^b^Overall hearing disability as calculated according to the Department of Health and Social Security (DHSS) formula: [(4 × better hearing ear) + (worse hearing ear)]/5

^c^Mean total COMQ-12 score excluding item 6

In the logistic regression analysis, there was a significant association between worse-ear hearing and dizziness (AOR 1.01; 95% CI 1.00–1.02; *p* = 0.0177). There was also a significant region effect, with study participants in European countries (AOR 1.53; 95% CI 1.03–2.28; *p* = 0.0344) and Colombia (AOR 2.48; 95% CI 1.25–4.92; *p* = 0.0096) more likely to report dizziness than participants in Asian countries. However, the presence of ear discharge and cholesteatoma were not associated with dizziness in the adjusted analyses (Table 2).

**Table 2:** Logistic regression analysis of factors associated with presence of dizziness reported by patients with chronic otitis media (*n* = 477)

Factors	Unadjusted OR (95% CI)	*p*	Adjusted OR (95% CI)	*p*
Worse-ear hearing	1.01 (1.00–1.02)	**0.0306**	1.01 (1.00–1.02)	**0.0177**
Ear discharge (present vs. absent)	1.49 (0.95–2.33)	0.0801	1.30 (0.75–2.22)	0.3510
Cholesteatoma (present vs. absent)	1.01 (0.65–1.59)	0.9510	0.95 (0.55–1.67)	0.9163
European countries vs. Asian countries	1.74 (1.22–2.48)	**0.0206**	1.53 (1.03–2.28)	**0.0344**
Colombia vs. Asian countries	3.33 (1.88–5.89)	** < 0.0001**	2.48 (1.25–4.92)	**0.0096**
Colombia vs. European countries	1.92 (1.10–3.30)	**0.0192**	1.62 (0.83–3.14)	0.1563

Significant *p* values are highlighted in bold

*CI* confidence interval, *OR* odds ratio

## Discussion

In the present study, over half of participants with COM (56.2%) reported at least mild inconvenience related to dizziness or disequilibrium. This was similar to participant responses in previous studies utilizing the COMQ-12 questionnaire [11, 15, 16, 19] and other studies reporting the prevalence of vestibular symptoms in patients with COM (44–59.5%) [20–24]. Although participants could reasonably have other causes for their vestibular symptoms unrelated to COM, our results suggest that dizziness is a common burden in this population, which warrants further enquiry.

Based on data from a longitudinal, population-based cohort, Aarhus et al. [25] demonstrated that childhood chronic suppurative otitis media (CSOM) and childhood hearing loss secondary to recurrent acute otitis media (AOM) are associated with an increased risk of dizziness in adulthood compared to adults with normal childhood hearing and a negative history of recurrent AOM. There is mounting histopathological evidence supporting the hypothesis that disease chronicity and severity of middle ear inflammation are crucial factors influencing the development of long-term inner ear deficits, including sensorineural hearing loss, tinnitus, and balance disorders [10]. Recent studies have shown that the density of loss of vestibular (and cochlear) hair cells in human temporal bones from donors who had CSOM was more severe than in human temporal bones from donors who had serous or purulent otitis media [9, 10]. Labyrinthine fistula is a recognized complication of COM (incidence ranging from 3.6–12.9%), most commonly affecting the lateral semicircular canal, leading to sensorineural hearing loss, dizziness, and vertiginous attacks [26]. In the absence of fistulae, penetration of bacterial toxins and inflammatory mediators via the permeable round window membrane to the inner ear has been proposed as a likely pathophysiological mechanism, causing direct injury to the labyrinth, local inflammation, and development of endolymphatic hydrops [27–29].

The major finding of this large observational study is that worse-ear hearing was identified as a risk factor for dizziness and disequilibrium in patients with COM. This is consistent with previous clinical observations [20, 22], however the strongest evidence for combined cochlear-vestibular impairment secondary to COM comes from histopathological studies as described earlier [9, 10]. Abnormalities in vestibular function tests are often detected in patients with COM who do not complain of vertigo/dizziness [8]. Furthermore, Mostafa et al. identified a significant association between duration of disease and a history of vertigo [23]. Taken together, it would appear that inflammatory damage to the inner ear is a chronic, slow process, thus allowing for adequate central compensation in daily situations. In such cases, subclinical vestibular deficits would only manifest symptomatically in challenging environments (e.g., disequilibrium in a dark environment) or during an acute episode of active middle ear inflammation [8, 20, 22]. Our findings suggest that patients with a greater degree of hearing impairment in the worse hearing ear are more likely to experience dizziness or balance problems. We hypothesize that this could represent a threshold effect, whereby the inflammatory disease processes have caused sufficient inner ear injury, with associated deterioration in vestibular function, such that central compensation is no longer adequate. Such deficits are likely to be permanent.

We identified an association between geographical location and prevalence of vestibular symptoms. Study participants in Colombia were more likely to report dizziness or disequilibrium than participants in both Asian and European countries, although the latter association was not significant in the adjusted model. In low to middle-income countries, COM is the most frequent cause of persistent mild to moderate hearing loss among young populations [30]. In Colombia, based on a representative sample of 200 adult patients with a diagnosis of COM, a recent analytical study reported that an average of 12% of household monthly income is spent on COM-related costs (often expenses related to transportation to specialist health centers) and disease duration exceeds 26 years on average [31]. This reflects the economic burden of COM in low to middle-income countries and suggests that patients are unable to access timely surgical intervention. This is consistent with our hypothesis that, irrespective of current middle-ear status (active vs. inactive), disease chronicity may increase the risk of developing long-term inner ear deficits. We also observed that participants in European countries were more likely to report dizziness than participants in Asian countries. In the present study, both geographical regions consist of a majority of countries with high-income economies (*Europe*: France, Italy, UK; *Asia*: Japan, South Korea). Cultural differences may account for regional variation in how patients assess their disease-specific health when completing patient-reported instruments. Due to limited number of centers in each country, we are unable to comment on intraregional variation in presentation of dizziness.

To our knowledge, the present observational study comprises the largest sample of patients with a diagnosis of COM to assess both the prevalence of and potential risk factors for dizziness. Participants were recruited from nine otology units across eight countries with wide heterogeneity in clinical presentation and disease activity, as well as including patients who had undergone middle-ear surgery previously. Therefore, by utilizing a prospective dataset, we hope to minimize the risk of selection bias and ensure the real-world applicability of our findings.

However, our study has several limitations. The presence of dizziness and/or disequilibrium was determined by participant responses to a single item within the COMQ-12 questionnaire, which is a crude form of assessment. Dizziness is a common complaint in the general population [5] and represents a spectrum of symptoms, often unrelated to peripheral vestibular pathology. Notably, isolated hearing loss is associated with reduced postural control, affecting balance and increasing the risk of falls [32, 33]. Patients are unlikely to differentiate true vertigo from dizziness, which usually requires thorough clinical assessment. Ideally, one should evaluate vestibular function formally to provide clinical correlation for patient-reported symptoms [8]. Several vestibular tests, such as the video head impulse test (vHIT) and bone-conduction vestibular evoked myogenic potential (BC-VEMP), bypass the middle ear, are noninvasive and could feasibly be integrated within a vestibular clinic appointment as part of pre-operative assessment or serial monitoring [34]. However, as the present study represents a secondary analysis of a prospectively obtained dataset, collecting further follow-up data was not possible. It is also worth considering that specialist balance testing often produces unreliable results [35] and is unlikely to identify subtle abnormalities in vestibular function secondary to inner ear injury due to inflammatory disease processes. We were unable to determine whether patient co-morbidity, other disease-related characteristics (e.g., duration, unilateral vs. bilateral), and gender influence the presentation of dizziness in COM. Furthermore, in the original study [18], ear discharge was determined by clinician-reported otoscopy during a single consultation to provide an objective and consistent form of assessment. Whilst this provides a useful snapshot of disease activity, unlike patient-reported assessment, otoscopy is inherently limited in evaluating patients with chronic or persistent drainage. This may account for the lack of association between ear discharge and dizziness in the present analysis. Finally, pure-tone audiometry was limited to air conduction testing only. Therefore, we were unable to evaluate the degree of air–bone gap or comment specifically on the association between sensorineural hearing loss and presence of vestibular symptoms.

The COVID-19 pandemic has caused significant disruption to elective surgical care and healthcare services globally are struggling to manage the huge backlog of patients requiring surgery [36]. There is an unmet need to identify suitable methods of surgical prioritization based on clinical urgency. As per issued guidance in the United Kingdom, the Federation of Surgical Specialty Associations recommends that procedures for cholesteatoma and suppurative otitis media should be performed in < 3 months and > 3 months, respectively [37]. Considering our findings and that of other studies [20, 22], future work should seek to determine whether a specific value for mean hearing thresholds across a range of frequencies in the affected ear(s) could predict which patients are most likely to suffer long-term vestibular sequelae secondary to COM. This would provide a transparent method and clinical justification for stratifying patients with non-cholesteatomatous middle ear disease for surgical intervention.

## Conclusion

The validated COMQ-12 questionnaire is a useful screening tool for common symptoms and sequelae of COM. Consistent with previous reports, our study demonstrates that vestibular symptoms frequently contribute to burden of disease in patients with chronic middle ear disease. We also found a significant association between hearing disability in the worse hearing ear and presence of vestibular symptoms. Geographical variation in presentation of dizziness may reflect financial barriers to treatment or cultural differences in how patients reflect on their health state.

## Supplementary Information

Below is the link to the electronic supplementary material.

Supplementary file1 (PDF 43 KB)
