# Supplementary material for: Presentation of dizziness in individuals with chronic otitis media: data from the multinational collaborative COMQ-12 study
Source: Eur Arch Otorhinolaryngol. 2021 Jul 21;279(6):2857–63. doi: 10.1007/s00405-021-06993-1 (PMC8294833; doi:10.1007/s00405-021-06993-1)
Supplement: Supplementary file 1 — Supplementary file1 (PDF 43 KB) [file 405_2021_6993_MOESM1_ESM.pdf]

Chronic Otitis Media Questionnaire - 12 (COMQ-12)

Authors: Phillips J, Yung M, Haggard M

These questions are to find out how badly your ear problems affect you. No machine can do this: only you can tell us. We expect the results from this questionnaire to help us understand which of your ear symptoms is the most important to you. Knowing this will help us improve the ways patients with ear problems are looked after.

Please answer the questions below by considering carefully each question asked, and then ringing the appropriate number; the numbers each refer to a particular description. There are no right or wrong answers, but please try to think carefully about each question before ringer the appropriate number. Please consider each problem as it has been over the past six months.

EXAMPLE:

For the following question, please indicate how often you perform this activity using the scale below and by ringing the appropriate number:

0 Never
1 At least once every 3 months
2 At least once every month
3 At least once a week
4 Most days in the week
5 All the time

How often do you eat toast for breakfast? **0 1 2 3 (4) 5**

A person responding like this conveys (s)he usually has toast but not always

For the following questions, please indicate <u>how severe</u> the various elements described affect you, using the scale below and by ringing the appropriate number:

- *0 Doesn't bother me at all*
- *1 A minor inconvenience*
- *2 A moderate inconvenience*
- *3 A major inconvenience but I can cope*
- *4 A major inconvenience and I am finding it hard to cope*
- *5 The worst thing that has ever affected my life*

Symptom severity:

1.	Discharge or drainage from the ear	0	1	2	3	4	5
2.	Having a 'smelly ear'	0	1	2	3	4	5
3.	Hearing problems at home, e.g. requiring the volume of the TV or Radio to be turned up.	0	1	2	3	4	5
4.	Hearing problems when talking to people in groups or when there are noisy surroundings	0	1	2	3	4	5
5.	Discomfort in and/or around the ear	0	1	2	3	4	5
6.	Dizziness or feeling 'off balance'	0	1	2	3	4	5
7.	Tinnitus or noises in the ear	0	1	2	3	4	5

For the following questions, please indicate how often the various elements described affect you using the scale below and by ringing the appropriate number:

- *0 Less frequent than once every 6 months*
- *1 At least once every 6 months*
- *2 At least once every 3 months*
- *3 At least once every month*
- *4 At least once a week*
- *5 Most days in the week*

Lifestyle and work impact:

How often have you NOT been able to:

8.	Perform your normal daily activities at home / work?	0	1	2	3	4	5
9.	Wash or shower or bathe as you would like to? i.e how often have you been fearful of these activities causing an ear infection?	0	1	2	3	4	5

Health service impact:

10.	How often have you been to see your GP about your ear problems ?	0	1	2	3	4	5
11.	How often do you need to take medicines (including eardrops) for your ear problem ?	0	1	2	3	4	5

For the following question, please indicate how bad things are, on a scale of '0' to '5'. '0' means not at all, and '5' means the worst you can ever imagine:

General:

12.	To what degree do your ear problems 'get you down'?	0	1	2	3	4	5

Please check that you have produced an answer to every question and do ask for help if you find it hard.

- Thank you very much for taking part.
